# Editorial

**Published:** 1864

**Authors:** 


### Editorial.

Expanation.—The desire to issue the whole of the excellent report of Dr. Prince in one number of the Examiner, has caused us to delay the August number, and issue it and the September number together. All the remaining papers and reports read to the recent meeting of the Illinois State Medical Society, will appear in the October number of the Examiner; after which we shall resume the *Clinical* department as formerly.

Suspension of Medical Journals.—Since the commencement of the rebellion, many of our most valuable medical periodicals have been discontinued, and still others seem to be in a wavering condition.

The most recent suspension is that of the *American Medical Times*, published in New York City. We think such a result is hardly creditable to the profession of that great City and State. War or no war; high prices or low prices; the profession should sustain, at least, one substantial medical periodical in New York. So far as the Examiner is concerned, its subscription list is steadily increasing, with greater promptness in payment than at any previous period. Yet we have never wrote a *dunning* line to our subscribers, nor uttered a word of complaint on account of high prices or rebellious times. The only difficulty we encounter is to find time to perform our editorial work promptly and properly.

Medical Colleges in Chicago.—The two medical schools in this city are already beginning to receive students for the ensuing lecture term. The annual lecture season in the Rush Medical College commences on Wednesday, October 5th, and continues sixteen weeks. At the close of their last annual session it was intimated, if not distinctly promised, that important improvements should be made in the college building; and that greater facilities for hospital clinical instruction should be provided, before the opening of the next annual lecture term. We see no indications that such changes have been made. On the contrary, the lecture rooms and clinical privileges seem to remain the same as last year. A general lecture term of *sixteen weeks;* with one or two dispensary clinics and one hospital clinic each week, comprises the whole of their annual college course.

The Annual Winter Course or lecture term in the Chicago Medical College (Med. Dept. of Lind University) will commence on Monday, October 10, 1864, and continue until the first Tuesday in March.

The lecture rooms, library, museum, and laboratory are all in excellent order; and beside a full course of five months in the college, the connection with the Mercy Hospital is such, that the senior students are enabled to attend one clinic at the bedside every day. The Clinical Course embraces three lectures each week in the medical department, one of which will be on diseases of the eye and ear; two in the surgical department, and one in the obstetric, relative to diseases of women and children.

---

### THE CASE OF SURGEON GENERAL HAMMOND.

---

Some time since we announced that Dr. Hammond was on trial before a duly constituted military commission at Washington, and although that trial has been the subject of a great many newspaper comments and dispatches, we have foreborne any remarks on the subject, preferring to await the slow process and decision of the authorities, which after so long a time has been made public. The following report of the Judge Advocate gives the charges and specifications, with the findings of the court and the approval of the President. We publish the entire report for the information of our readers. In addition we see it stated in the newspapers that by the order of the Secretary of War, Mr. Solicitor Whiting is directed to prosecute Ex-Surgeon-Gen. Hammond, Messrs. Wythe & Brothers, and Wm. A. Stephens, for the recovery of $450,000 said to be the amount of which Government was defrauded by the contracts specified in the charges below.

The Court was composed as follows:—

Major-General R. J. Oglesby, U.S.V. President; Brigadier-

General W. S. Harney, U.S.A.; Brigadier-General W. J. Ketchum, U.S.V.; Brigadier-General G. S. Green, U.S.V.; Brevet Brigadier-General W. W. Morris, Colonel 2d U.S. Artillery; Brigadier-General A. P. Howe, U.S.V.; Brigadier-General J. P. Slough, U.S.V.; Brigadier-General H. E. Paine, U.S.V.; Brigadier-General J. C. Starkweather, U.S.V.; Major John A. Bingham, Judge Advocate.

JUDGE ADVOCATE GENERAL'S OFFICE, MAY 17, 1864.

*To the Honorable Secretary of War:*—

Brigadier-General William A. Hammond, Surgeon-General United States Army, was tried upon charges of "disorders and neglects, to the prejudice of good order and military discipline," "conduct unbecoming an officer and a gentleman," and "conduct prejudicial to good order and military discipline."

The specifications which set forth the statement of facts alledged, and found by the court to constitute these offences, are as follows:—

CHARGE 1ST.—"Disorders and neglects, to the prejudice of good order and military discipline."

*Specification* 1st. "In this: that he, Brigadier-General William A. Hammond, Surgeon-General United States Army, wrongfully and unlawfully contracted for, and ordered Christopher C. Cox, as acting purveyor in Baltimore, to receive blankets of one William A. Stephens, of New York. This done at Washington City, on the seventeenth day of July, in the year of our Lord one thousand eight hundred and sixty-two.

*Specification* 2d. "In this: that he, Brigadier-General William A. Hammond, Surgeon-General, as aforesaid, did, on the thirtieth day of May, in the year of our Lord one thousand eight hundred and sixty-three, at Washington City, wrongfully and unlawfully prohibit Christopher C. Cox, as medical purveyor for the United States in Baltimore, from purchasing drugs for the army in said city of Baltimore."

*Specification* 3d. "In this: that he, the said Brigadier-General, William A. Hammond, Surgeon-General United States Army, did unlawfully order and cause one George Cooper, then medical purveyor for the United States, in the city of Philadelphia, to buy of one William A. Stephens, blankets, for the use of the Government service, of inferior quality: he, the said Brigadier-General William A. Hammond, then well knowing that the blankets so ordered by him to be purchased as aforesaid were inferior in quality, and that said purveyor Cooper had refused to buy the same of said Stephens. This done at Philadelphia, in the State of Pennsylvania, on the twenty-eighth day of May, in the year of our Lord one thousand eight hundred and sixty-two."

*Specification* 4th. "In this: that he, the said Brigadier-General William A. Hammond, Surgeon-General as aforesaid, on the fourteenth day of June, in the year of our Lord one thousand eight hundred and sixty-two, at the city of Washington, in the District of Columbia, unlawfully, and with intent to aid one William A. Stephens to defraud the Government of the United States, did, in writing, instruct George E. Cooper, then medical purveyor at Philadelphia, in substance as follows:

"'Sir.—You will please purchase of Mr. W. A. Stephens eight thousand pairs of blankets, of which the inclosed card is a sample. Mr. Stephens' address is box 2500, New York. The blankets are five dollars per pair.'"

*Specification* 5th. "In this: that he, the said Brigadier-General William A. Hammond, Surgeon-General United States Army, on the sixteenth day of June, in the year of our Lord one thousand eight hundred and sixty-two, at the city of Washington, did corruptly, and with intent to aid one William A. Stephens to defraud the Government of the United States, give to the said William A. Stephens an order in writing, in substance as follows: 'Turn over to George E. Cooper, medical purveyor at Philadelphia, eight thousand pairs of blankets;' by means whereof the said Stephens induced said Cooper, on Government account, and at an exhorbitant price, to receive of said blankets, which he had before refused to buy, seventy-six hundred and seventy-seven pairs, and for which the said Stephens received payment at Washington, in the sum of about thirty-five thousand three hundred and fourteen dollars and twenty cents."

*Specification* 6th. "In this: that he, the said Brigadier-General William A. Hammond, Surgeon-General United States Army, on the thirty-first day of July, in the year of our Lord eighteen hundred and sixty-two, at the city of Philadelphia, in the State of Pennsylvania, well knowing that John Wyeth & Brother had before that furnished medical supplies to the medical purveyor at Philadelphia, which were inferior in quality, deficient in quantity, and excessive in price, did corruptly, unlawfully, and with intent to aid the said John Wyeth & Brother to furnish additional large supplies to the Government of the United States, and thereby fraudulently to realize large gains thereon, and then and there give to George E. Cooper, medical purveyor at Philadelphia, an order, in writing, in substance as follows:"

"'You will at once fill up your store-houses, so as to have constantly on hand hospital supplies of all kinds for two hundred thousand men for six months. This supply I desire that you will not use without orders from me.'"

"And then and there direct said purveyor to purchase a large amount thereof, to the value of about one hundred and seventy-three thousand dollars, of said John Wyeth & Brother."

*Specification* 7th. "In this: that he, the said Brigadier-General William A. Hammond, Surgeon-General United States Army, about the eighth day of October, in the year of our Lord eighteen hundred and sixty-two, at Washington City, in contempt of, and contrary to the provisions of the act entitled 'An act to recognize and increase the efficiency of the medical department of the army,' approved April 16, 1862, did unlawfully direct Wyeth & Brother, of Philadelphia, to send forty thousand cans of their 'extract of beef' to various places, to wit: to Cincinnati, St. Louis, Cairo, New York, and Baltimore, and send the account to the Surgeon-General's office for payment."

CHARGE 2D.—"Conduct unbecoming an officer and a gentleman."

*Specification* 1st. "In this: that he, Brigadier-General William A. Hammond, Surgeon-General United States Army, on the thirteenth day of October, in the year of our Lord eighteen hundred and sixty-two, at Washington City, in a letter by him then and there addressed to George E. Cooper, declared in substance that, the said Cooper had been relieved as medical purveyor in Philadelphia, because, among other reasons, 'Halleck,' meaning Major-General Henry W. Halleck, General-in-Chef, requested as a particular favor that Murray might be ordered to Philadelphia; which declaration so made by him, the said Brigadier-General William A. Hammond, Surgeon-General, as aforesaid, was false."

An additional charge and specifications preferred against Brigadier-General William A. Hammond, Surgeon-General United States Army:

CHARGE 3D.—"Conduct to the prejudice of good order and military discipline."

*Specification* 1st. "In this: that he, the said Brigadier General William A. Hammond, Surgeon-General United States Army, on the eighth day of November, in the year of our Lord eighteen hundred and sixty-two, at Washington City, did unlawfully order Henry Johnson, then medical storekeeper and acting purveyor at Washington City, to purchase three thousand blankets of one J. P. Fisher, at the price of $5.90 per pair, and to be delivered to Surgeon G. E. Cooper, U.S.A., medical purveyor at Philadelphia."

A plea of not guilty was entered upon each of the charges and specifications, and after a full hearing of the testimony for the Government and the defence, and the examination of a large amount of documentary evidence, together with the consideration of the elaborate arguments of both sides, the court rendered a finding of guilty on all the charges, and sentenced the accused to be dismissed the service, and to be forever disqualified from holding any office of honor, profit, or trust, under the Government of the United States.

In reporting upon this case, the second charge—conduct unbecoming an officer and a gentleman—will be first considered.

Under this charge it was alleged that accused made a false declaration, in writing, that Dr. Cooper had been relieved from his position as medical purveyor at Philadelphia, because among other reasons, General Halleck had requested as a special favor, that Dr. Murray might be ordered to duty in that city.

It appears from the evidence that, on the 8th of October, accused requested of the Adjutant-General that Dr. Cooper be relieved from duty as medical purveyor, at Philadelphia, by Dr. Smith. On the 13th, he wrote a letter to Dr. Cooper, as follows:—

"My Dear Doctor.—I have just received your note. The detail for your relieval from duty went to the Adjutant-General a few days since. I told Smith to tell you of it. It was with great reluctance, even with pain, that I made the detail. I am entirely satisfied with your energy, faithfulness, and acquaintance with your duty; but I found great complaints made in regard to your manners, which were constantly reiterated from medical officers and citizens of standing. I believe the change would have been made over my head had I not made it myself. I was forced to come to the conclusion that it was necessary to be done. Once before the detail was made, but I would not sign it, and this time it lay on my table several days. This is one reason. The second is even more imperative. Halleck requested, as a particular favor, that Murray might be ordered to Philadelphia. There was nothing for Murray to do there but to take your place, King's, or Smith's. The latter have both been in active service, and I thought it best to relieve you on that account.

"As A. K. Smith is, in my opinion, better suited to perform the duties of purveyor than Murray, I decided to make him purveyor, and Murray medical director of transportation.

"I assure you that, so far as your official action is concerned, I have not the least fault to find. Yours sincerely,

W. A. HAMMOND."

General Halleck testified, substantially that "to the best of his recollection," he never made any request of the accused to order Dr. Murray to Philadelphia; the only communication he ever made to him on the subject being a letter on the first of October, stating that Dr. Murray had served long and faithfully in the field, with the army in the West, and would like to be transferred to Eastern hospital duty, and asking a consideration of his case.

On the part of the defence, a letter from Dr. Murray to General Halleck, dated Louisville, September 27th, was submitted, in which Dr. Murray stated to General Halleck, that if he would request the Surgeon-General to order him to Philadelphia, it would "be done at once." And it was claimed by the accused—but not shown—that General Halleck, besides writing the letter of October 1st, in which he asked that Dr. Murray's desire to be ordered East on "hospital duty" might be considered, also, in some personal interview, made a verbal request that he be assigned to that duty in Philadelphia.

The argument of the Judge Advocate on this charge may be found on pages 57 to 59 of his "Reply," and that of the counsel for the accused on pages 51 to 53 of the "Defense."

The findings upon the first and third charges involve questions of law as well as of fact.

It was contended by the accused (see pages 9 and 16 of the "Defence") that the Surgeon-General had the power to control all purchases of stores for his department; to order purveyors when, at what places, of whom, and at what prices they should procure them; and further, that he might purchase them himself.

It was submitted by the Judge Advocate (see pages 4 to 7 of his "Reply") that the Acts of Congress of April 16, and July 17, 1862, limited the authority of the Surgeon-General to the direction when to purchase, and the kind and quantity to be procured; that having given this direction, his lawful authority was determined, leaving to medical purveyors, under bonds for the proper discharge of their responsibilities, the whole duty of selecting in such markets, and of buying from such persons, and upon such terms as their judgment dictated.

The former of these enactments provides "that medical purveyors shall be charged, under the direction of the Surgeon-General, with the selection and purchase of all medical supplies, including hospital stores," etc., etc.

The latter makes provision that medical purveyors shall give bond, with approved security, in such sums as the Secretary of War shall require, for the faithful performance of their duties.

It would seem, from the express language as well as from the reason of the law, that the position taken by the Judge Advocate was correct, and the decision of the court upon this issue was warranted. But it is deemed unnecessary to bestow further consideration upon this question. The findings of the court, that the accused ordered the purveyors to purchase supplies of inferior quality, well knowing them to be such, and to purchase articles at exorbitant prices, with corrupt intent to aid in defrauding the Government, and that he ordered the purchase of "additional large supplies," "corruptly," and "with intent to aid" certain persons "fraudulently to realize large gains thereon," impute much more than a mere technical overstepping of the limits of the enactment of April 16, 1862. They convict him of official corruption, abuse of power, and a gross breach of public trust.

The proof upon which these finding are based was offered in support of the 3d, 4th, 5th, 6th, and 7th specifications to the first charge. It is not requisite in this report to collate and comment upon it. The full presentation of the whole case by the Judge Advocate relieves this office of the necessity of entering into that detailed discussion of the facts and legal questions involved which, under different circumstances, would have been proper.

In his "Reply," and the "Defence" of the counsel for the accused, both of which are printed and attached to the record, the important portions of the evidence and all the prominent features of the proceedings, are presented as concisely as the voluminous character of the testimony would admit.

That the natural and necessary result of the acts of the accused as established by the record, involved a criminal spoilation of the Government treasury, which would alone have called for his dismissal from the service, cannot be denied; but when it is remembered, as shown by the proof, that this spoilation was in part accomplished by the purchase of inferior medical supplies and stores—thus compromising the health and comfort, jeopardizing the lives of the sick and wounded soldiers in the hospitals and upon the battle fields of the country—soldiers solemnly committed to the shelter and sympathies of the office held by the accused, by the very law and purpose of its creation—it must be admitted that this fearfully augments the measure of his criminality.

The trial, which lasted nearly four months, was one of the most patient and thorough that has ever occurred in our military history; and the accused had throughout the assistance of eminent and able counsel in conducting his defence. The court, which was composed of nine general officers, at the close of this prolonged investigation, declared him guilty of the charges preferred, and awarded the punishment which, in their judgment, was in accordance with the nature and degree of the offences committed; and a careful examination of the record leaves no room for doubt as to the validity of the proceedings, or the justness of the findings and sentence.

J. HOLT, *Judge Advocate General.*

The following is the President's order confirming the sentence in this case:—

"The record, proceedings, findings, and sentence of the court in the foregoing case are approved; and it is ordered that Brigadier-General William A. Hammond, Surgeon-General of the United States Army, be dismissed the service, and be forever disqualified from holding any office of honor, profit, or trust under the Government of the United States.

A. LINCOLN.

*August* 18, 1864."—*Cincinnati Lancet and Observer.*

